# Explicit Configurational Entropy of Mixing in Molecular Dynamics Simulations

**DOI:** 10.1021/acs.jpclett.4c02819

**Published:** 2024-11-05

**Authors:** T. Hanke, A. L. Upterworth, D. Sebastiani

**Affiliations:** Department of Chemistry, Martin Luther University, 06120 Halle, Germany

## Abstract

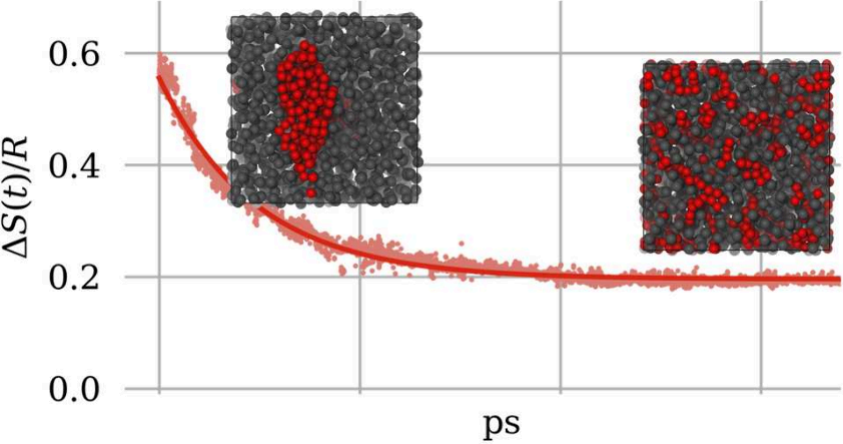

The entropy of mixing of a multicomponent system of particles is a simple expression of the molar fractions for the equilibrium state, but its intermediate values for transient (nonequilibrium) states can not be calculated directly from the particle coordinates so far. We propose a simple expression for the configurational entropy of mixing based solely on the set of instantaneous coordinates, which is suitable for the on-the-fly determination of the degree of mixing along a molecular dynamics trajectory. We illustrate the applicability of our scheme with the example of several molecular mixtures that exhibit fast and slow mixing and demixing processes within a molecular dynamics simulation.

Entropy is the basic thermodynamic driving force, complementary to the enthalpy, which determines the spontaneous evolution of all processes in nature. As one of the most fundamental thermodynamic quantities, it is relevant in all areas of natural sciences. Within “molecular” chemistry, some of the many relevant applications of entropy as a thermodynamic driving force are solution processes and phase transitions/phase equilibria. Traditionally, the entropy is defined either via its total differential as the reversibly exchanged reduced heat, *dS* = *δQ*_rev_/*T*, or on the absolute scale via the statistical weight of a macrostate, *S* = *k*_*B*_ ln Ω. One of the elementary processes driven directly by entropy is the mixing of noninteracting particles of two species *A* and *B*, starting from two separated pure phases. The difference in Entropy between the mixed and separated state is commonly known as entropy of mixing, Δ*S* = *S*(mixed) – *S*(separated). In a system composed of *N*^(*A*)^ particles of species *A* and *N*^(*B*)^ particles of species *B*, the molar entropy of mixing is given as
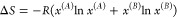
1with the molar fractions *x*^(λ)^ = *N*^(λ)^/(*N*^(*A*)^ + *N*^(*B*)^) and the universal gas constant *R*. eq 1 constitutes the equilibrium value of the entropy of mixing for a fully mixed phase, and is therefore not directly applicable to nonequilibrium states of a system. A formally very similar idea in the field of information theory was pioneered by Shannon^1^ in view of quantifying the amount of information on a data stream.

Surprisingly, no explicit and simple way has been published so far to express the entropy of mixing of a system of several components based on the instantaneous particle coordinates directly, i.e. in a form that allows a direct measure of the temporal evolution of the configurational entropy of mixing during a simulation. While the topic of the entropy of mixing has attracted considerable interest over recent years, all existing approaches are based on the starting point from statistical thermodynamics. The entropy is expressed as an absolute function via either the statistical weight or the partition function of a configuration, yielding the entropy of mixing as the differences of the corresponding absolute values of pure and actual phases. An excellent recent work by Desgranges^2^ illustrates the principal power of this idea; they used a Monte Carlo approach to compute the statistical weight Ω, and further used an extended Wang–Landau approach to compute partition function of binary mixtures.^3^ The pioneering work on this fundamental path was done by Lazaridis^4,5^ and Berens,^6^ and further developed by Peter.^7^ A particular aspect of this approach is that it can be formulated using binary correlation functions to express the entropy of mixing. For a truly ergodic system, such correlation functions could be computed using one single configuration, but for most actual simulations, an ensemble average at equilibrium would be required. A related yet complementary interesting approach is the *k*^*th*^ nearest neighbor method of entropy estimation, recently reviewed by Fogolari et al.^8^ This concept is equally able to quantify the configurational entropy on the basis of a Shannon-type expression ρ(**r**)*ln*(ρ(**r**)). There is actually a certain disagreement in the literature about the question whether the conventional expression *x* ln(*x*) or the Flory–Huggins formula *x* ln(*V*) is more accurate for computing the entropy of mixing of actual polymers; according to Lazaridis, the conventional expression turns out to yield better results.^4,5^ A complementary idea was followed by Garces,^9^ who determined clusters of particles in the mixture and derived an entropy expression from their probabilities. One of the more fundamental approaches was developed by Lin,^10^ based on computing the partition function of a system via its vibrational density of states from the atomic velocity autocorrelation functions, and deriving the entropy of mixing from the partition function. This approach was applied successfully^11^ and extended further by Caro.^12^ Detmar et al.^13^ found, that the displacement parameter of a Monte Carlo simulation is linearly tied to the residual entropy of a binary mixture. This makes the estimation of entropy exceedingly simple for Monte Carlo simulations, under the common condition that their phase space sampling is converged - in other words, at equilibrium. In order to show the validity of their method, Detmar et al. used Widoms insertion^14^ to calculate the chemical potential and from that the residual entropy of the system. A more frequently used analysis tool to describe the chemical environment in molecular simulations are pair correlation functions (also known as radial distribution functions). Minimum distance distribution functions, a special type, have recently been exploited to quantify the local structure around complex solutes and accumulation or exclusion characteristics.^15^ A complementary mainly experimental tool are partition coefficients which measure the concentration ratio of a solute between two solvents.^16^ These partition coefficients can be determined directly from molecular simulation.^17,18^ An interesting recent development by Brehm^19^ is based on a related expression for microheterogeneities via histogram-based evaluation of local density fluctuations. Economou used them for the determination of Gibbs energies of solvation^20^ and Huyskens was able to derive a closed expression for the partition coefficients of alkanes/alkanoles in water based on thermodynamic considerations of mobile disorder.^21^ It should be noted that this topic is of high industrial relevance, there is a well established annual competition organized by industry (“Industrial Simulation Challenge”^22^), where properties of fluids like the heat of mixing, liquid–liquid interfaces and partition coefficients of unknown systems are predicted by several simulation teams.^23,24^

However, while all these approaches are of course valid and useful, they are not directly applicable to a given moment within a molecular simulation for answering the simple question whether the components are well mixed or not, i.e. whether the entropy of mixing of a particular set of configurations is rather close to zero (separated state) or close to the maximum value (corresponding to full mixing), given by the expression in eq 1.

In this letter, we propose a novel scheme for the explicit calculation of the instantaneous entropy of mixing of a molecular system based directly on the atomic coordinates. The scheme is simple and fast, and does not require any level of additional simulation (such as the calculation of partition functions, autocorrelation functions, or variations of lattice occupation numbers).

To illustrate the idea of our scheme, we start by a model system of a large number of red and blue particles distributed randomly in a box (see Figure 1).

**Figure 1:**
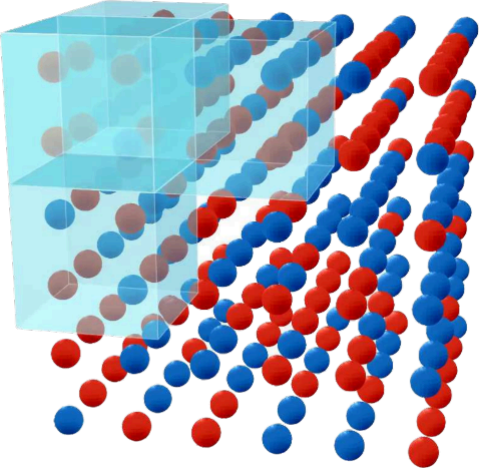
Model system consisting of a binary mixture of particles (red and blue). The total box is split into small elementary volumes *V*_α_ (blue boxes).

The total volume can be divided into *N* small elementary volumes *V*_α_ (blue boxes in Figure 1) which are assumed to contain a sufficiently large number of particles. As entropy is an extensive quantity, the total entropy of mixing of the entire system Δ*S* can be written as the sum over the entropies of mixing Δ*S*_α_ of the elementary volumes α. Now, we assume that the individual elementary volumes are sufficiently small, so that each subsystem can be considered to be in local equilibrium, which in turn enables the application of eq 1 for each individual elementary volume *V*_α_:
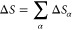
2
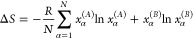
3

Here, *x*_α_^(*A*)^ is the molar fraction of species *A* in the elementary volume element *V*_α_. If all elementary volumes have the same molar fractions *x*^(*A*)^ and *x*^(*B*)^, then eq 3 evolves to eq 1, i.e. the system is at equilibrium (fully mixed). In the other extreme of fully separated phases, all elementary volumes will have *x*_α_^(*A*)^=1, *x*_α_^(*B*)^=0 or vice versa, leading to Δ*S* = 0. Hence, this expression captures the essential features of the instantaneous entropy of mixing in a multicomponent system, i.e. eq 3 is able to describe the evolution from nonequilibrium to equilibrium states numerically.

While this model derivation is in principle valid, it is difficult to apply it to real-world simulations, since the basic assumptions (large total number of particles, large number of elementary volumes, sufficiently large number of particles in each elementary volume) can normally *not* be satisfied simultaneously.

Our novel scheme starts here. Instead of repartitioning the total volume in finite elementary volumes with constant molar fractions *x*_α_^(*A*)^ and *x*_α_^(*B*)^, we switch to defining *molar fraction distributions x*^(*A*)^(**r**) and *x*^(*B*)^(**r**). A reproduction of the model system (Figure 1) would be achieved by defining *x*^(*A*)^(**r**∈*V*_α_)=*x*_α_^(*A*)^. Instead, we define the molar fraction distributions based solely on the partial densities of the two species ρ^(*A*)^(**r**) and ρ^(*B*)^(**r**) via
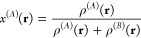
4

The key is now to define the partial densities ρ^(*A*)^(**r**) and ρ^(*B*)^(**r**) via the actual atomic coordinates of the particles. To this aim, we replace each particle *i* by a density distribution localized around the coordinate of the particle **R**_*i*_. We have chosen to use a Gaussian function according to
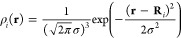
5
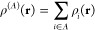
6with a specific broadening parameter σ which will be discussed later. In this equation, the particle coordinates **R**_*i*_ can be taken directly from a single frame of a molecular dynamics trajectory, i.e. the resulting molar fraction distributions *x*^(*A*)^(**r**) and *x*^(*B*)^(**r**) in eq 4 are actually functions of the simulation time. Now it is straightforward to generalize the entropy of mixing Δ*S*_α_ of an elementary volume element α to a global entropy distribution Δ*S*(**r**) according to
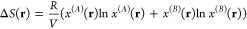
7

This entropy distribution is a continuous function defined throughout the simulation box, and based on the instantaneous particle coordinates **R**_*i*_. The total entropy of mixing of the entire system is then given as
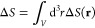
8

This integral is computed using a numerical integration library (integrals.jl^25^ making use of the HCubature^26^ algorithm), a Julia implementatiton of a multidimensional integration package with an adaptive local resolution. The integration is performed for each neighboring pair of particles and can be restricted to a very local environment, since the integrand is of very small support (either the partial molar fractions *x*^(*i*)^(**r**) or their logarithm quickly decay to zero).

This explicit expression for the entropy of mixing Δ*S* of a molecular system, eq 8 in combination with eq 4, 5, and 6, represents the essence of our letter. Our scheme provides a simple, fast, and direct approach to compute the entropy of mixing based on the instantaneous atomic coordinates **R**_*i*_(*t*) along the trajectory of a molecular dynamics simulation run. All other thermodynamic effects on the entropy (such as the entropy variation due to changes in pressure/volume/vibrational frequencies/···) are automatically excluded from this expression; its numeric range is limited to the interval [0; Δ^(theo)^*S*] with the theoretical equilibrium entropy of mixing Δ^(theo)^*S* according to eq 1. While we have limited our derivation to two different species, the generalization to an arbitrary number of different compounds is straightforward.

Note that the derivation presented here contains a “free” parameter σ, corresponding to the Gaussian broadening of each particle. The actual broadening could of course be realized by other suitable functions, such as exponentials, Lorentzian functions, error functions or also Heaviside functions. The choice of the broadening function shape and the broadening parameter σ will have a certain influence on the numerical results from our approach; here we have chosen to stick to the simplest solution, i.e. Gaussian broadening and a broadening coefficient σ chosen close to the van-der-Waals-radius of the corresponding atom. The effect of this choice is presently investigated in more detail and will be published soon.

We have illustrated the individual steps to follow within our scheme for the calculation of the entropy of mixing for two species in Figure 2, using the example of a one-dimensional chain of particles, which are placed at integer coordinates. Two distinct model situations are generated, i.e. with two distinct particle arrangements (left vs right column in Figure 2). The first situation (left) represents two pure phases with a localized interface at coordinate 4.5 (arbitrary units). The second situation (right) represents a gradually more mixed phase.

**Figure 2:**
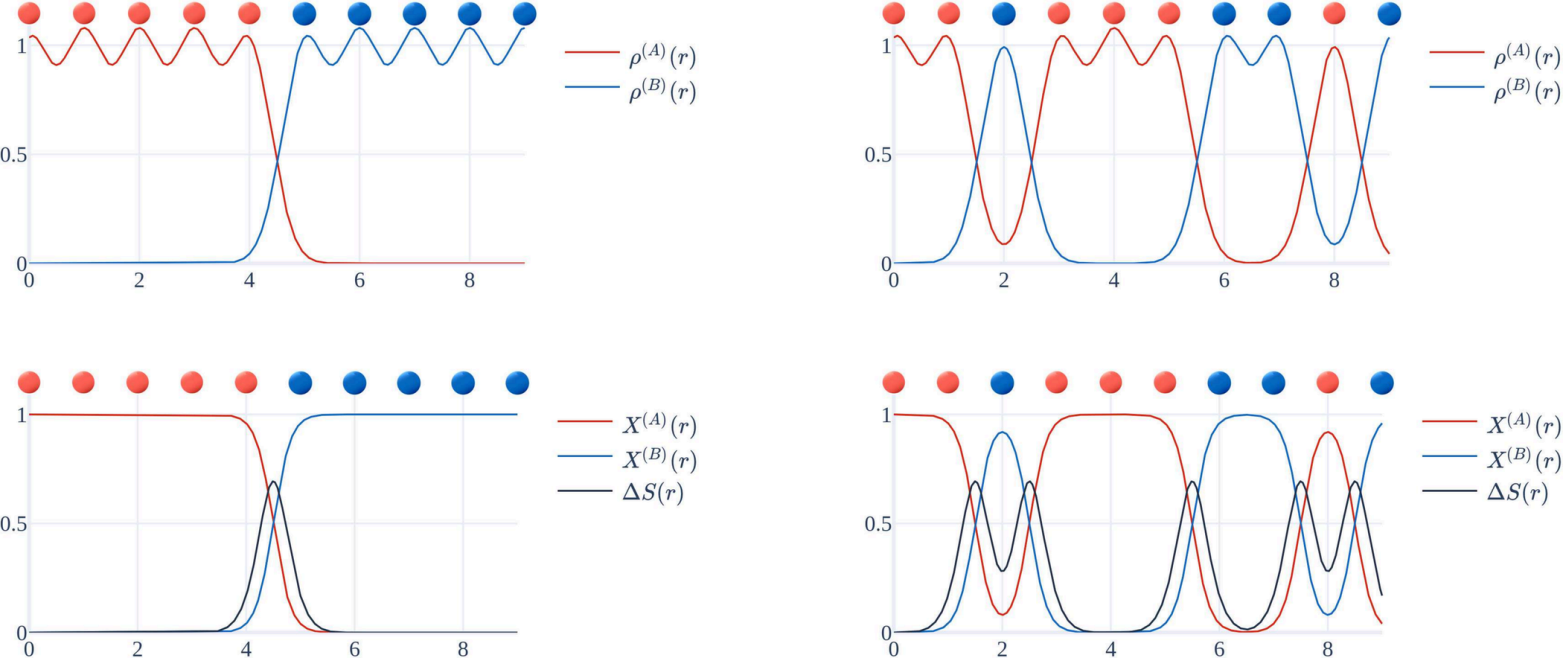
Illustration of the individual steps of our scheme. Shown is the transition from two distinct arrangements (left vs right plots) of particles (red/blue spheres, placed at integer coordinates) to broadened particle densities (ρ^(*A*)^(**r**), ρ^(*B*)^(**r**), top left and top right), then to molar fraction distributions (*x*^(*A*)^(**r**), *x*^(*B*)^(**r**), bottom figures) and the resulting distribution of the entropy of mixing Δ*S*(**r**) (black line in the bottom figures). Only the interface area between the A and B species generates a sizable contribution to the entropy distribution. Note that for clarity Δ*S*(**r**) is plotted in units of *R*/*V*.

The entropy distribution resulting from the application of our scheme, eq 4-8is shown as the black function in Figure 2. Clearly, only the respective interface regions between red and blue particles generate a significant contribution to the entropy distribution. Already two adjacent particles of the same species result in a vanishing molar fraction density of the other species and thus to a vanishing entropy distribution in that area. Alternating –A–B–A–B– arrangements, however, result in a molar fraction density oscillating around 0.5 and thus in a larger entropy distribution.

The entropy of mixing is maximum for a perfectly mixed state. In our model system with *N*^(*A*)^ = *N*^(*B*)^, this corresponds to an alternating particle arrangement, –A–B–A–B–, with an entropy of mixing of Δ*S* = *R* ln 2 according to eq 1. The maximum value that our expression for the entropy of mixing may achieve is slightly lower than this theoretical value. The reason for this is that the Gaussian delocalization of each particle results in molar fraction densities oscillating around 0.5 and thus an entropy distribution somewhat below the ideal limit. The magnitude of this effect can be controlled via the particle broadening according to eq 5: the more the individual particles are delocalized, the more the molar fraction distribution will approach a value of 0.5.

Note that the precise shape of all functions shown here depends to some degree on the broadening function and the broadening parameter σ. This more technical aspect will be addressed in a forthcoming publication. However, we have observed that the qualitative results for the entropy distribution only depend weakly on these parameters. An additional interesting point is the reaction of our entropy distribution to a spatial separation of the two phases. It turns out that the contribution of the interface region to the entropy of mixing is considerably reduced, but does not vanish. This point is illustrated in the Supporting Information.

Our goal is to provide an efficient tool for the characterization of liquid mixtures. We have thus applied eq 8 to an actual molecular dynamics simulation, specifically a binary water/methanol mixture () at *T* = 300 K. The simulation was started with completely separated phases (see the illustration in Figure 3 and converged to a visually well mixed state at around 500 ps simulation time. During this mixing process, we have computed the instantaneous entropy of mixing (see Figure 3) for every 100th trajectory step (0.1 ps). The initial value of the entropy of mixing of 0.2 *R* for the separated phases reflects the finite size of our simulation box: While two strictly separated phases of infinite size would yield a value of exactly Δ*S* = 0, the layered structure of our computational setup implies a considerable “mixing” of the two species already in the initial “separated” state.

**Figure 3:**
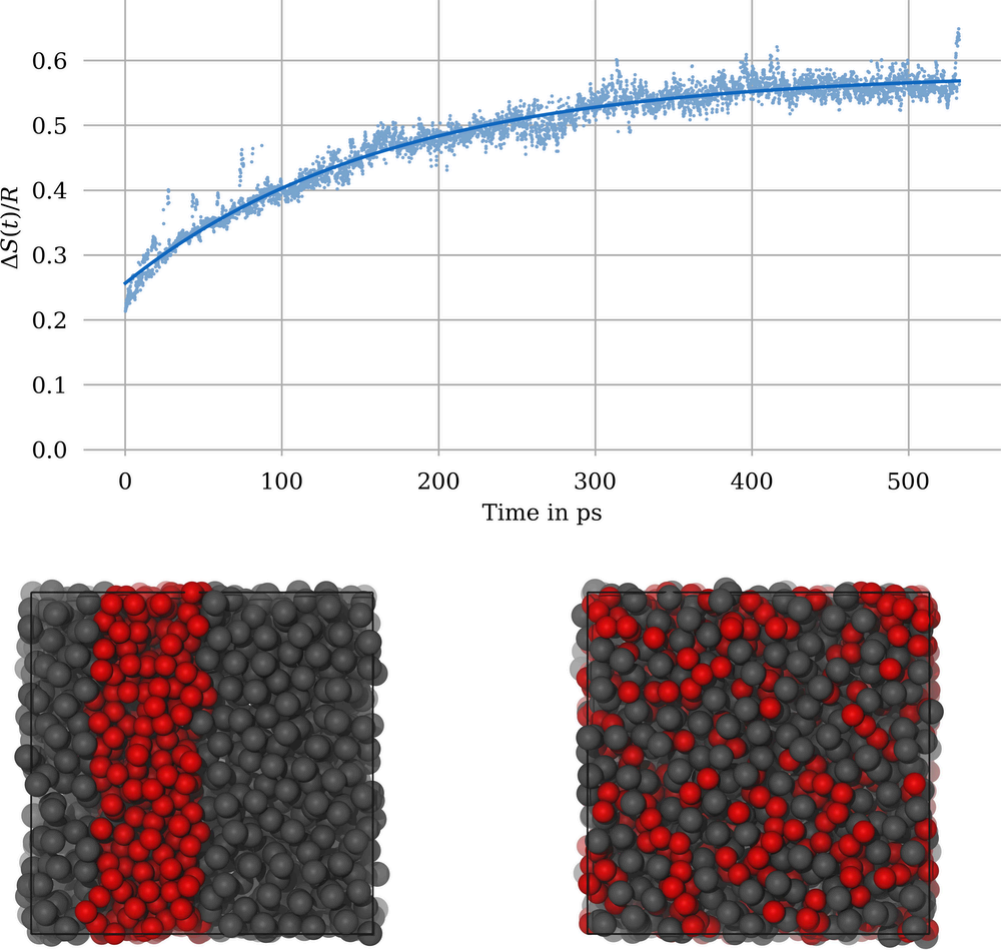
Evolution of the entropy of mixing during the mixing process of water and methanol at 300 K (solid line: exponential fit). Snapshots of the simulation box at *t* = 0 ps and *t* = 500 ps are shown below (gray, methanol; red, water). Note that Δ*S*(*t*) is plotted in units of *R*.

During the MD simulation, the diffusion of water and methanol molecules leads to visually observable configurations of increasingly mixed state. Our expression for the entropy of mixing is nicely able to quantify this increase in mixing, as illustrated in Figure 3. The evolution of the entropy of mixing shows a certain amount of numerical noise, but can be fitted to an exponential function Δ*S*(*t*) = Δ*S*_0_ + Δ*S*_*∞*_(1–exp(−*t*/τ)), which converges to Δ*S*_0_ + Δ*S*_*∞*_ = 0.58 *R*. The theoretical value for the equilibrium entropy of mixing of Δ^theo^*S* = *R* ln 2 ≈ 0.69 *R* is not fully reached. This reflects the limits of our numerical scheme, in particular the effect of the finite broadening parameter σ in eq 5. A large value for σ would result in a closer agreement between Δ*S*_0_ + Δ*S*_*∞*_ and Δ^theo^*S*, but at the cost of a higher initial entropy value in the separated state. Even for a perfectly mixed molecular configuration, our molar fraction densities *x*^(*A*)^(**r**) and *x*^(*B*)^(**r**) exhibit peaks (and minima respectively) at the molecular coordinates, which has been illustrated in Figure 2. Consequently, Δ*S* deviates numerically from the theoretical value. The optimal choice for the broadening parameter σ is to some degree system-depedendent and must be adjusted individually for a given simulation. We are presently investigating the dependence of Δ*S*_0_ and Δ*S*_*∞*_ on the choice of σ, in view of providing a practical guide for the optimal numerical choice of this parameter.

Another question is to which extent the system size influences our numerical results. We have therefore performed a simulation of the same water–methanol system with a 8-fold larger system. The resulting entropy evolution is shown in Figure 4. It turns out that the numerical value for Δ*S* converges at virtually the same value as for the smaller system (0.54*R*), illustrating a very modest system size dependence of our approach. The fact that the value is still below the equilibrium value of an ideally mixed system corresponds to the tendency of this particular system to exhibit local density fluctuations, i.e. regions/clusters with increased methanol concentration. This partial microphase separation could be confirmed visually by inspecting the trajectory.

**Figure 4:**
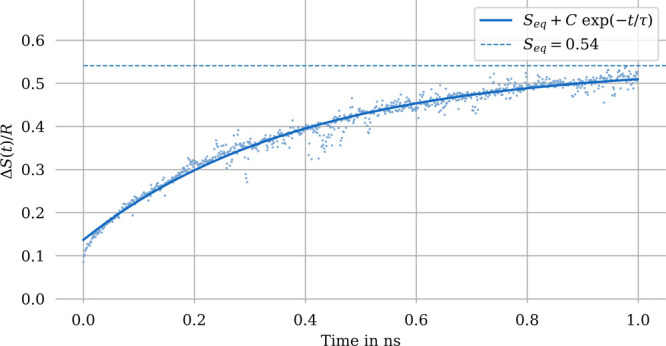
Entropy of mixing (water and methanol at 300 K) for an 8-fold larger system compared to Figure 3.

Complementary to the mixing process (of water and methanol), we have also studied the demixing process of two immiscible liquids, here water and trichloromethane at 300 K. The simulation was started from a random distribution of the molecules in the simulation box, while all other settings were kept the same as in the previous mixing simulation. Figure 5 shows the evolution of the entropy of mixing computed along the entire molecular dynamics trajectory according to eq 8. At the initial mixed state, the entropy is high and close to its theoretical maximum of Δ*S* = *R* ln 2. As trichloromethane and water molecules diffuse and gradually form separated phases, the entropy of mixing decreases exponentially and converges to 0.2 *R* after about 0.5 ns. This value is the same as for the separated state of water and methanol (see Figure 3). Again, the theoretical limit of Δ*S* = 0 is not reached due to the finite size of the two phases. The exponential fit yields a characteristic demixing time of τ ≈ 170 ps, which reflects the strongly immiscible nature of these two solvents.

**Figure 5:**
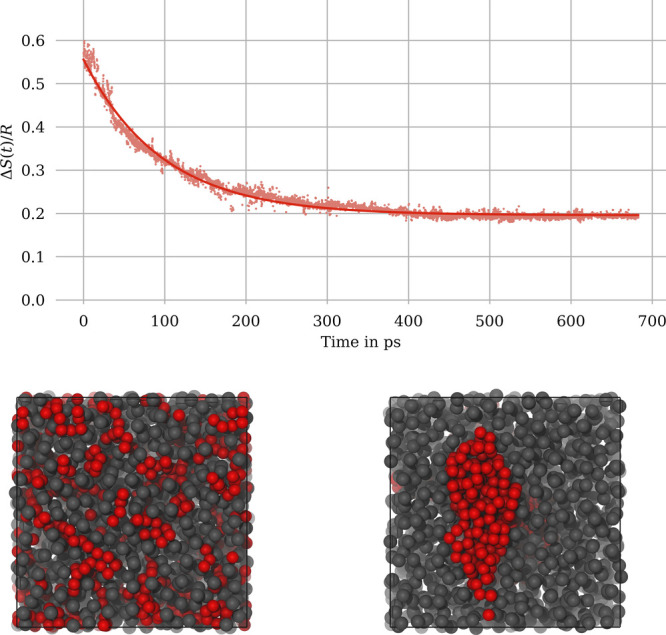
Evolution of the instantaneous entropy of mixing during the demixing process of water and trichloromethane at 300 K, along with an exponential fit, all in units of *R*. Snapshots of the simulation box at the beginning and end of the demixing process are also shown (red, water; gray, trichloromethane).

Also in this case, we have checked the dependence on system size by repeating the water–trichloromethane demixing simulation for an 8-fold larger system (results shown in Figure 6). It turns out that the mixing entropy of the demixing process converges to a lower final value for the larger system. This behavior is expected, since under periodic boundary conditions, the smaller system corresponds to the larger system in a state with eight trichloromethane clusters, hence a lesser degree of phase separation.

**Figure 6:**
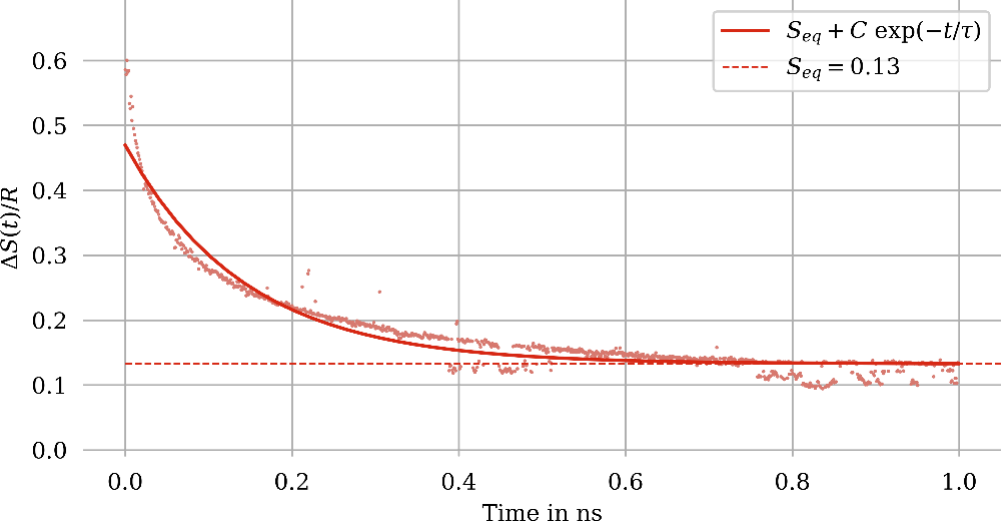
Entropy of demixing (water–trichlormethane, at 300*K*) for an 8-fold larger system compared to Figure 5.

In both the mixing and the demixing processes, entropy fluctuations by up to 0.1 *R* are observed at time scales of few femtoseconds. These are caused by translational and rotational motions of individual molecules in the simulation trajectory, where even small geometric changes can influence the instantaneous entropy value. These fluctuations are analogous to those of other observables (e.g., the potential energy) during an MD simulation. We are presently investigating amplitude and frequency spectrum of these entropy fluctuations and their relation to system size, particle size and temperature in more detail. It is an interesting question whether these entropy fluctuations can be exploited within the fluctuation–dissipation theorem for the characterization of a canonically conjugated observable; formally, this observable should be the temperature itself (since ).

Beyond the validation of our expression for the entropy of mixing for these two pairs of elementary solvents, we have applied our scheme to a more complex system with a less obvious behavior, specifically a hexane/perfluorohexane mixture. Perfluorinated molecules constitute an own class of philicity, complementary to the better known hydrophilicity and lipophilicity categories.^27−32^ Experimentally, this binary system is mixed at temperatures above the upper critical solution temperature (UCST) of 296 K and phase-separated below.^33−35^ From a simulation perspective, we have chosen both a temperature close to the UCST (*T* = 300 K, initially phase-separated) as well as a significantly lower one (*T* = 200 K, initially mixed) in order to see how the entropy of mixing describes these situations. Figure 7 shows characteristic snapshots of our molecular dynamics simulations along with the evolution of Δ*S*(*t*), again in units of R for the sake of clarity.

**Figure 7:**
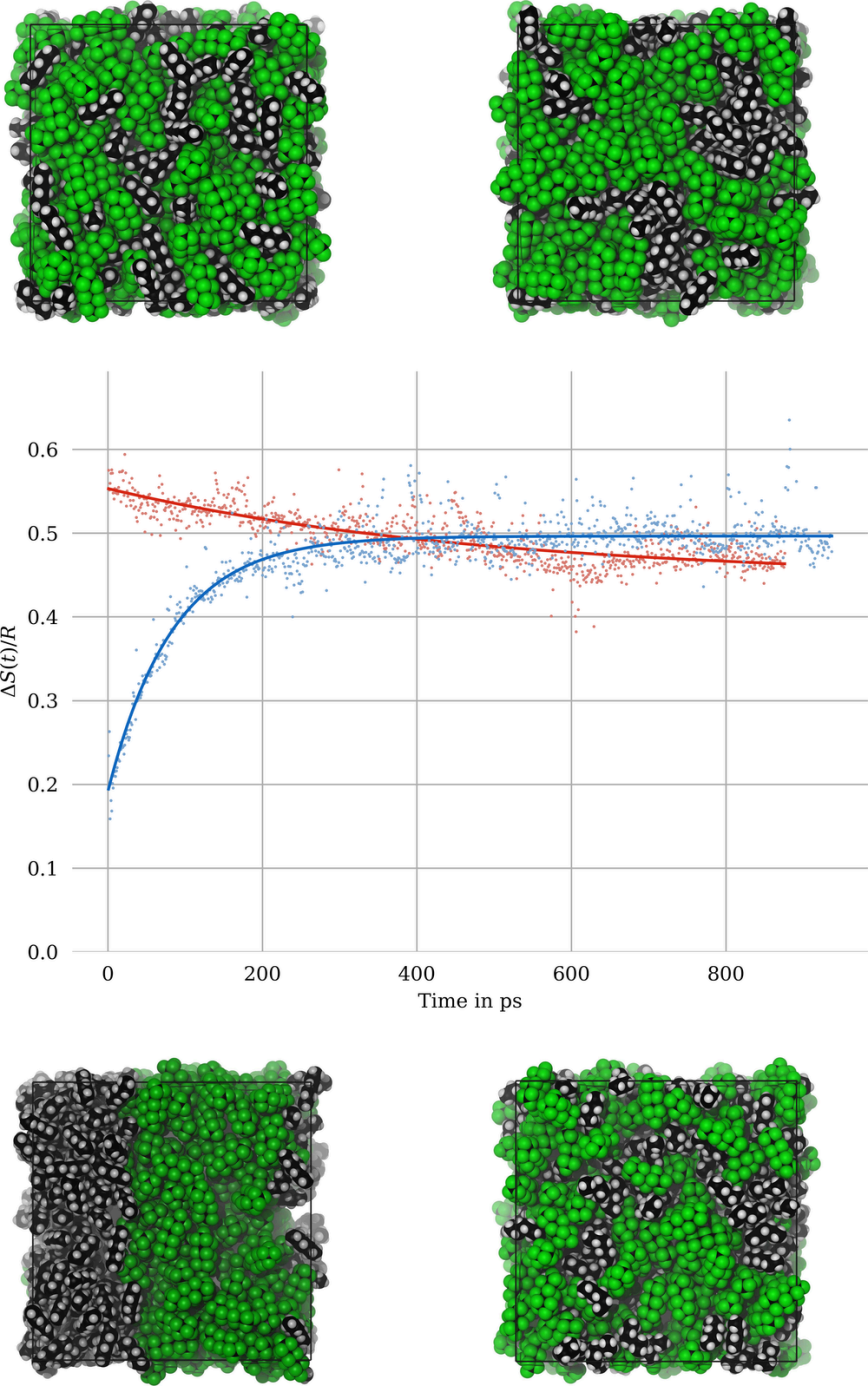
Evolution of the entropy of mixing during the molecular dynamics simulations of hexane and perfluorohexane (with exponential fits, Δ*S* in units of *R*). Trajectory snapshots are shown for the initial configurations as well as for *t* ≈ 1 ns at *T* = 200 K (top) and *T* = 300 K (bottom).

The evolution of the entropy of mixing of the hexane/perfluorohexane mixture for both temperatures is shown in Figure 7. The blue curve (T = 300 K, initially phase-separated) shows a straight exponential increase from a typical phase-separated entropy value of 0.2 R, and converges to 0.5 R, which is somewhat below the theoretical limit of 0.69 R, and also below the value that we obtained previously for the mixtures of small solvents (water, methanol). We believe that this deviation is most likely due to the proximity of our simulated temperature (*T* = 300 K) to the UCST (experimentally, *T*_UCST_ = 296 K) and a thus a consequence of incomplete mixing. The characteristic time scale of mixing is about τ = 100 ps. The illustrations of the simulation boxes (images below the plot) show the transition from a fully separated to a mostly mixed phase, confirming the numerical results for Δ*S*.

The entropy of mixing at the lower temperature (red curve in Figure 7) starts at an initially higher entropy than the converged value for the phase-separated system at *T* = 300 K and decreases slowly. The corresponding characteristic time scale of demixing is therefore quite large and can only be estimated from the present simulation (τ ≈ 10 ns), also because there is a considerable amplitude of numerical noise. Interestingly, the initial entropy of mixing (Δ*S*(*t* = 0) ≈ 0.55 R) is almost identical to the values obtained in the simulations of the much smaller molecular systems (water, methanol, trichloromethane). This illustrates the colligative nature of the entropy of mixing.

The images with the conformational snapshots for the *T* = 200 K simulation (top of Figure 7) correspond to the structures at *t* = 0 and *t* = 1 ns. A slight tendency for the onset of phase separation is visible, but this tendency is difficult to describe other than qualitatively. Our method for the quantitative characterization of the entropy of mixing, however, allows for an explicit numerical description of the state of (de)mixing, which is inaccessible to the eye. It should be noted that despite the slow decay of Δ*S*(*t*), the entropy change of about 0.1 *R* after 1 ns corresponds to about 25% of the total variability between fully mixed and fully separated phases. This illustrates the potential for characterization of our entropy expression for complex molecular systems.

In conclusion, we have developed and validated a general-use expression for the entropy of mixing of a molecular system of several components. The expression is straightforward to implement and will soon be available for routine analysis within established simulation packages.^36^ A quantitative description (with physical units) of the instantaneous degree of mixing during a molecular simulation is achieved while only atomic coordinates are required for the evaluation of the entropy. It should be noted that partial density fluctuations and binary particle correlations are not explicitly needed as functions, but they are incorporated indirectly through the particle coordinates and their evolution. We have introduced a spatial broadening parameter which has a minor numerical influence and can be adjusted to the molecular size of the particles. Our approach is therefore applicable to any nonequilibrium situation and can be used to characterize the time scale of entropic processes, but of course also to benchmark the convergence of a simulation from an ergodicity perspective. Possible applications include simple and complex liquids such as solutions of ions and molecules, but also liquid/liquid and liquid/solid interfaces (including e.g. protein/water/salt interfaces) where it corresponds to the interfacial entropy.

A natural perspective of our entropy expression is the characterization of solubilities and partition coefficients, with particular focus on the concept of (poly-) philicity,^27−32^ but also the solution of small atoms/molecules in complex liquids and their structural/dynamical effects such as the Hofmeister series^37,38^ and the understanding of the solubility of “difficult” solutes such as cellulose.^39−42^

## Data Availability

The code for computing the instantaneous entropy of mixing for the systems used here can be downloaded free of charge from the Github repository https://github.com/tillhanke/mixingentropy.

## Supporting Information Available

Computational details and illustration of the effect of spatial separation of the two phases on the entropy distribution (PDF)
