# Supplementary material for: Explicit Configurational Entropy of Mixing in Molecular Dynamics Simulations
Source: J Phys Chem Lett. 2024 Nov 5;15(45):11320–7. doi: 10.1021/acs.jpclett.4c02819 (PMC11571226; doi:10.1021/acs.jpclett.4c02819)
Supplement: Supplementary file 1 — jz4c02819_si_001.pdf [file jz4c02819_si_001.pdf]

Supporting Information
Explicit Configurational Entropy of Mixing in Molecular Dynamics Simulations

T. Hanke, A. L. Upterworth, and D. Sebastiani*

Department of Chemistry, Martin Luther University, 06120 Halle, Germany

E-mail: daniel.sebastiani@chemie.uni-halle.de

Spatial separation of pure phases

We have studied one particular interesting feature of our scheme for the calculation of the entropy of mixing on the example of the model system of red/blue point particles. Specifically, we have created a certain spatial separation between the two pure phases, and computed the resulting change in the partial particle densities, the molar fraction distributions and the entropy distribution. The resulting graphs are shown in Fig. S1

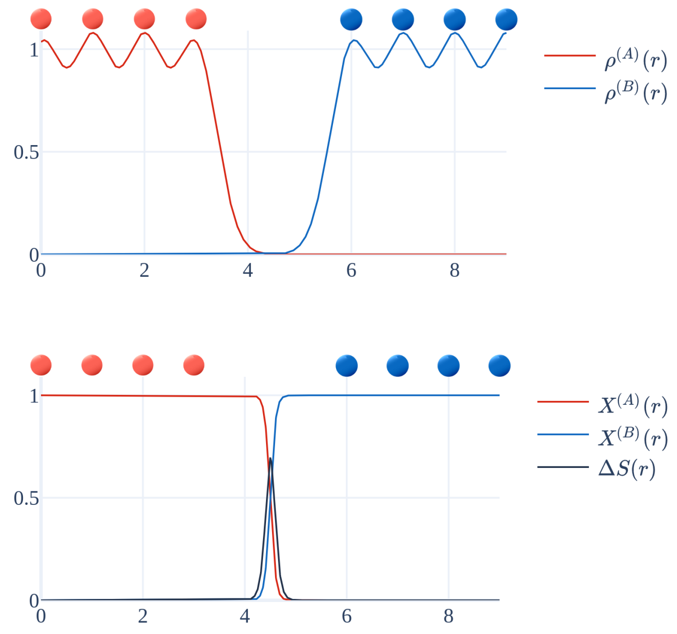

Figure S1: Top: exemplary partial density $\rho(\mathbf{r})$; bottom: entropy distribution $S(\mathbf{r})$ (black) and molar fraction distribution $X(\mathbf{r})$. The two different types of particles are visualised in red or blue. Here we visualise again a totally separated system as in Fig. 2 (left) with the key difference of having moved the phases further away from each other. This results in a larger gap between the particles on the separating positions.

The partial densities show the expected decay to zero, creating a region of nearly zero total density around coordinate value 4.5. As the densities are constructed from Gaussian functions, they are never exactly zero, so that the molar fraction distributions $x^{(A)}$ and $x^{(B)}$ remain well-defined (bottom plot). As it turns out, the most significant change is their sharper transition from 1 to 0 and vice-versa, which in turn reduces the spatial region where the entropy distribution is non-vanishing. Hence, the primary effect of this spatial

separation is to lower the effective entropy of mixing. This result is completely consistent with the intuitive expectation that a "cleaner" separation of two pure phases reduces the entropy of the system.

Three dimensional example states

We have taken a intermediate-sized system of point particles of two species, and we have computed the entropy of mixing in (a) the fully phase-separated state, (b) the perfectly mixed state (represented by the rock-salt crystal structure, i.e. all A particles on a cubic lattice with all B particles on the same cubic lattice but displaced by the vector (a/2,a/2,a/2), and (c) a state generated with random particle coordinates. The resulting entropies of mixing (in units of R) are 0.02, 0.63, and 0.46 , which means that for this model system, we perfectly reach virtually quantitatively the analytically expected values. The corresponding calculations can be viewed in the examples directory.

Computational Details

Implementation of the algorithm was done with the JuliaLang[1] programming language while making use of the Integrals library.[2] The final code can be found in the Github repository under `https://github.com/tillhanke/mixingentropy`.

For the graphic representations we used the following python[3] libraries:

- MANIM,[4] a community driven animation engine for the 3D graphics in the introduction
- Plotly,[5] a interactive graphing library
- matplotlib,[6] another graphing library

All molecular dynamics (MD) simulations were performed using the LAMMPS[7,8] software package and a time step of 1 fs. An OPLS-aa (Optimized Potentials for Liquid Simulations

all-atom) type potential[9] was used with OPLS-aa parameters for methanol,[9] trichloromethane,[9] hexane[9] and perfluorohexane.[10] Water was represented by the TIP4P-EW[11] model in the simulation with trichloromethane and by the SPC/E[12] model in the one with methanol and the aqueous hydrogen atoms were constrained by the SHAKE[13] algorithm. Short-range Lennard-Jones and Coulombic interactions were calculated up to a cut-off radius of 800 pm. For long-range electrostatic interactions the pppm (particle-particle particle-mesh) solver[14] was used. In general, the geometric mean rule was applied for mixed pairwise interactions with exception of the fluorine-hydrogen interaction, for which a special combination rule developed by Morgado et al.[15] was applied.

Simulation boxes were prepared with PACKMOL.[16] In order to visualize mixing processes, molecule types were packed into two separated slabs, whereas for demixing processes all molecules were distributed randomly over the whole simulation box. In the case of methanol-water and trichloromethane-water, the simulation boxes contain 1000 molecules of each kind. For the larger system hexane-perfluorohexane the number of molecules was reduced to 250 each. Densities were determined in preceding equilibration runs consisting of multiple consecutive steps. Following energy minimization and velocity initialization according to the Maxwell distribution, an initial 25 ps long run in the NVE ensemble with direct temperature rescaling was performed. Afterwards, the ensemble was first changed to NVT for 50 ps and then to NpT for another 50 ps. To dampen shock waves resulting from the change in system size Langevin dynamics with a coupling constant of 100 fs were run for 50 ps followed by a longer run of 3.5 ns in the NpT ensemble where the density was averaged over the last 2.5 ns. Temperature and pressure control were achieved by Nosé-Hoover thermostats[17–19] and barostats with coupling constants of 100 fs and 2000 fs. Resized boxes matching the determined densities were then used to simulate both the mixing of methanol-water and the demixing of trichloromethane-water at 300 K as well as the mixing (at 300 K) and demixing (at 200 K) of hexane-perfluorohexane. After a 500 step energy minimization and velocity initialization, production runs were performed for 1 ns in the NVT ensemble.

Temperature control was achieved by a Nosé-Hoover thermostat[17–19] with a coupling constant of 100 fs. Atomic coordinates were written to the trajectory file in every time step. Entropy of mixing was then calculated for every hundredth time step. Snapshots of the simulation box were created using VMD[20] and the Tachyon[21] renderer.

References

(1) Bezanson, J.; Edelman, A.; Karpinski, S.; Shah, V. B. Julia: A fresh approach to numerical computing. *SIAM Rev Soc Ind Appl Math* **2017**, *59*, 65–98.

(2) Rackauckas, C.; Nie, Q. DifferentialEquations.jl – a performant and feature-rich ecosystem for solving differential equations in Julia. *J. Open Res. Softw.* **2017**, *5*, Exported from https://app.dimensions.ai on 2019/05/05.

(3) Foundation, P. S. Python. 2001; `https://www.python.org`.

(4) Developers, T. M. C. Manim – mathematical animation framework. 2023; `https://www.manim.community/`.

(5) Inc., P. T. Collaborative data science. 2015; `https://plot.ly`, Place: Montreal, QC Publisher: Plotly Technologies Inc.

(6) Hunter, J. D. Matplotlib: A 2D graphics environment. *Comput Sci Eng* **2007**, *9*, 90–95.

(7) Plimpton, S. Fast parallel algorithms for short-range molecular dynamics. *J. Comp. Phys.* **1995**, *117*, 1–19.

(8) Thompson, A. P.; Aktulga, H. M.; Berger, R.; Bolintineanu, D. S.; Brown, W. M.; Crozier, P. S.; in 't Veld, P. J.; Kohlmeyer, A.; Moore, S. G.; Nguyen, T. D. et al. LAMMPS - a flexible simulation tool for particle-based materials modeling at the atomic, meso, and continuum scales. *Comput. Phys. Commun.* **2022**, *271*, 108171.

(9) Jorgensen, W. L.; Maxwell, D. S.; Tirado-Rives, J. Development and testing of the OPLS all-atom force field on conformational energetics and properties of organic liquids. *J. Am. Chem. Soc.* **1996**, *118*, 11225–11236.

(10) Watkins, E. K.; Jorgensen, W. L. Perfluoroalkanes: Conformational analysis and liquid-state properties from ab initio and Monte Carlo calculations. *J. Phys. Chem. A* **2001**, *105*, 4118–4125.

(11) Horn, H. W.; Swope, W. C.; Pitera, J. W.; Madura, J. D.; Dick, T. J.; Hura, G. L.; Head-Gordon, T. Development of an improved four-site water model for biomolecular simulations: TIP4P-Ew. *J. Chem. Phys.* **2004**, *120*, 9665–9678.

(12) Berendsen, H. J. C.; Grigera, J. R.; Straatsma, T. P. The missing term in effective pair potentials. *J. Phys. Chem.* **1987**, *91*, 6269–6271.

(13) Ryckaert, J.-P.; Ciccotti, G.; Berendsen, H. J. C. Numerical integration of the cartesian equations of motion of a system with constraints: Molecular dynamics of n-alkanes. *J. Comp. Phys.* **1977**, *23*, 327–341.

(14) Hockney, R. W.; Eastwood, J. W. *Computer simulation using particles*; CRC Press, 1988.

(15) Morgado, P.; Martins, L. F. G.; Filipe, E. J. M. From nano-emulsions to phase separation: Evidence of nano-segregation in (alkane + perfluoroalkane) mixtures using 129Xe NMR spectroscopy. *Phys. Chem. Chem. Phys.* **2019**, *21*, 3742–3751.

(16) Martínez, L.; Andrade, R.; Birgin, E. G.; Martínez, J. M. PACKMOL: A package for building initial configurations for molecular dynamics simulations. *J. Comput. Chem.* **2009**, *30*, 2157–2164.

(17) Nosé, S. A unified formulation of the constant temperature molecular dynamics methods. *J. Chem. Phys.* **1984**, *81*, 511–519.

(18) Nosé, S. A molecular dynamics method for simulations in the canonical ensemble. *Mol. Phys.* **1984**, *52*, 255–268.

(19) Martyna, G. J.; Klein, M. L.; Tuckerman, M. Nosé-Hoover chains: The canonical ensemble via continuous dynamics. *J. Chem. Phys.* **1992**, *97*, 2635–2643.

(20) Humphrey, W.; Dalke, A.; Schulten, K. VMD – visual molecular dynamics. *J. Mol. Graph.* **1996**, *14*, 33–38.

(21) Stone, J. An efficient library for parallel ray tracing and animation. M.Sc. thesis, Computer Science Department, University of Missouri-Rolla, 1998.
